# Predicting cancer risk using machine learning on lifestyle and genetic data

**DOI:** 10.1038/s41598-025-15656-8

**Published:** 2025-08-19

**Authors:** Mohamed Abdelmoaty Ahmed, Ahmed AbdelMoety, Asmaa Mohamed Ahmed Soliman

**Affiliations:** 1 Faculty of Medicine, Merit University, Sohag, Egypt; 2 Electrical Engineering Department, Faculty of Engineering, South Valley University, Qena, 83523 Egypt; 3 Public Health and Community Medicine Department, Faculty of Medicine, Assiut University, Asyut, Egypt

**Keywords:** Cancer prediction, Genetic risk, Lifestyle factors, Machine learning, Cancer, Computational biology and bioinformatics, Diseases, Health care, Medical research, Risk factors

## Abstract

Cancer remains one of the leading causes of mortality worldwide, where early detection significantly improves patient outcomes and reduces treatment burden. This study investigates the application of Machine Learning (ML) techniques to predict cancer risk based on a combination of genetic and lifestyle factors. A structured dataset of 1,200 patient records was used, comprising features such as age, gender, Body Mass Index (BMI), smoking status, alcohol intake, physical activity, genetic risk level, and personal history of cancer. A full end-to-end ML pipeline was implemented, encompassing data exploration, preprocessing, feature scaling, model training, and evaluation using stratified cross-validation and a separate test set. Nine supervised learning algorithms were evaluated and compared, including Logistic Regression (LR), Decision Tree (DT), Random Forest (RF), Support Vector Machines (SVMs), and several ensemble methods. Among these, Categorical Boosting (CatBoost) achieved the highest predictive performance, with a test accuracy of 98.75% and an F1-score of 0.9820, outperforming both traditional and other advanced models. Feature importance analysis confirmed the strong influence of cancer history, genetic risk, and smoking status on prediction outcomes. The findings highlight the effectiveness of boosting-based ensemble models in capturing complex interactions within health data and support their potential use in personalized cancer risk assessment. This research underscores the value of integrating genetic and modifiable lifestyle variables into predictive modeling to enhance early detection and preventive healthcare strategies.

**Supplementary Information:**

The online version contains supplementary material available at 10.1038/s41598-025-15656-8.

## Introduction

### Background and motivation

#### Importance of cancer prediction

According to predictions, 13.1 million deaths worldwide are expected to be attributable to cancer by 2030, making it one of the major causes of death^1^. Survival rates significantly improve when detected early, which is crucial for effective treatment. Predictive modeling can identify individuals at increased risk for adverse outcomes, potentially years before clinical symptoms manifest, allowing for preventive actions to be implemented while the total illness burden remains low^2^. For instance, breast and lung cancer outcomes are substantially better when detected early, highlighting the life-saving potential of predictive tools^3,4^. Thus, recognizing genetic and lifestyle factors linked to cancer risk is essential for prevention and resource allocation.

#### Challenges in current diagnostic methods

Conventional diagnosis methods are often based on invasive pathological examination, imaging methods or late-stage biomarkers that are expensive, time-consuming, and often incapable of detecting cancer at its earliest stages^5^. Further, the assumptions of linearity and independence among variables in many traditional statistical models can be restrictive as they are not always appropriate or applicable for complex and most non-linear health data^6^. Additionally, traditional methods suffer from False Negatives (FNs) and positives making it sometimes diagnosing missing, and sometimes causing psychological and physical stress to patients and their surroundings^7^. These limitations emphasize the need for risk prediction tools that are more accurate, interpretable, and scalable.

#### Motivation for computational and data-driven approaches

The explosion of electronic health records, wearable devices, and genomic databases have resulted large volumes of structured data available for prediction, but extracting value is challenging. Machine learning (ML), a remarkably effective tool for analyzing multi-dimensional, heterogeneous datasets, is uniquely suited to identifying patterns and relationships in risk factors that may be invisible to the human eye, a capacity that has quickly turned ML into a transformative tool for cancer risk assessment^8,9^. Algorithms based on ensemble, such as Random Forest (RF) and Categorical Boosting (CatBoost), which achieved high accuracy in risk-factor identification using lifestyle behaviors and genetic profiles^10,11^, as well in the presence of noise or unbalanced data, provide also a reasonable performance. Predictive systems, powered by data-driven models of individualized assessment, incorporating both genetic pre-dispositions and removable risk factors, could then be designed to inform personalized prevention strategies and improve clinical decision-making and patient outcomes.

### Role of machine learning in healthcare

#### Growing impact of machine learning in medical diagnostics

ML has been pervasive in change the healthcare sector rapidly by allowing the diagnosis of disease earlier and much faster and more accurately. Unlike traditional statistical models, ML has the potential to analyze large heterogeneous health data, including electronic health records, imaging, genomic and other laboratory tests, and patient history in order to find subtle patterns that precede disease manifestation. Such data-based techniques can improve diagnostic precision, minimize human negligence and facilitate clinical decision making contemporaneously^12^. ML models have been extensively used to detect conditions such as cardiovascular disease and diabetes by combining several health measures from various metrics including blood pressure, cholesterol, and glucose levels^13,14^. This development is encouraging a transition from reactive care to a proactive and preventive healthcare system.

#### Applications in related medical domains

In addition to cancer, ML has shown considerable efficacy in other areas, including diabetes and cardiovascular disease. In diabetes management, ML models have been employed to forecast illness onset, enhance treatment regimens, and assess the risk of complications. Neural networks and ensemble models have surpassed conventional diagnostic approaches in forecasting blood glucose irregularities and diabetic consequences^15–17^. In the realm of cardiovascular disease, ML has enhanced the precision of risk assessments by examining elements sometimes neglected in traditional evaluations, including hereditary factors and lifestyle markers^14^. These successful applications underscore the significance of ML in healthcare and its capacity to transform diagnostics for many diseases.

#### Recent advances in cancer prediction

Over the last few years, numerous advances have been made in the domain of gene selection and predicting the classification of cancer using ML and hybrid optimization algorithms. As an example, mRMR has been combined with Salp Swarm Optimization and Weighted SVMs to enhance the applicability of gene relevance and a high breast cancer classification accuracy of 99.62% was obtained^18^. Another approach used RNA-Seq gene expression and deep learning models augmented with Harris Hawk and Whale Optimization techniques, gaining an improved accuracy in early breast cancer diagnosis^19^. Likewise, mRMR and the Northern Goshawk Algorithm were used to maximize gene selection and produced competitive outcomes across microarray datasets^20^. Using a two-stage genetic filtration and refinement, a different approach that merges Mutual Information with the Particle Swarm-Optimization (MI-PSA) has found that the accuracy is furthermore increased, at 99.01%^21^. Elsewhere, the Kashmiri Apple Optimization and Armadillo Optimization hybrid methods also showed high potential when implemented with SVM, with an F1-score of 99.22% on breast cancer data^22^. An additional successful method combined Random Drift Optimization with XGBoost classifiers in a variety of cancers such as CNS and leukemia and improved the accuracy and F-measure of standard models^23^. In addition, some metaheuristic combinations (such as Harris Hawks and Cuckoo Search) were also investigated for gene selection since they provide consistent mechanisms with less computational effort in high-dimensional datasets^24^. The hybrid Sine Cosine and Cuckoo Search Algorithm (SCACSA) shown efficacy in feature selection for breast cancer datasets by leveraging mRMR filtering and SVM classification^25^. Along with these practical contributions, a specific review was conducted for nature-inspired algorithms in cancer prediction, which included highly effective strategies to accomplish dimensionality reduction of biomedical data^26^. Lastly, a systematic review summarized the state of art of ML tools for cancer classification, establishing an in-depth comparison among supervised, unsupervised, and reinforcement learning models and highlighting the power of ML to change clinical practice in precision oncology^27^.

### Problem statement

Cancer is a leading cause of morbidity and mortality globally, and early diagnosis is essential to enhance patient outcomes and make treatment less burdensome. Conventional diagnostic approaches depend on invasive methods and costly laboratory exams which might not be reachable for everyone, specifically in resource-limited areas^28–31^. In addition, the factors associated with cancer risk are multifactorial, growing from combinations of genetic susceptibility as wells as risk factors, for example, smoking, alcohol, physical activity, and body composition. However, clinicians face challenges in synthesizing these multidimensional data to identify individuals at high risk. This study seeks to fill this gap by utilizing ML methods to forecast cancer risk through a synthesis of lifestyle and genetic factors. This study examines the efficacy of various supervised learning models in accurately classifying patients as likely or unlikely to develop cancer by analyzing a dataset of 1,200 patient records, which includes features such as age, gender, Body Mass Index (BMI), smoking status, genetic risk levels, physical activity, alcohol consumption, and cancer history. The objective is to develop a transparent and dependable predictive system that aids healthcare professionals in risk assessment and decision-making, potentially facilitating earlier interventions and improved resource allocation in clinical practice.

### Research objectives

The primary goals of this study are systematically outlined in Table 1, encompassing a list of research objectives in building a ML-based system for predicting cancer risk. Each target is aligned with different points of the dataset with data exploration, data pre-processing, model evaluation, and user interface. This reflected process guarantees addressing the technical quality as well as the practical usability across the research activities.

**Table 1:** Research objectives of the cancer risk prediction system based on lifestyle and genetic data.

Objective No.	Objective statement	Targeted dataset features	Expected outcome
RO1	To investigate and assess the distribution and patterns of patient health and lifestyle characteristics associated with cancer.	Age, BMI, PhysicalActivity, AlcoholIntake, Gender, Smoking, CancerHistory, GeneticRisk	Thorough Exploratory Data Analysis (EDA) plots and a correlation matrix to ascertain significant affecting factors.
RO2	To clean, standardize, and preprocess the dataset for optimal model training.	All features (excluding Diagnosis)	Dataset that is appropriately scaled and meticulously formatted for a ML workflow.
RO3	Multiple categorization models are trained and cross-validated.	All input features	Identifying top models using mean accuracy and standard deviation.
RO4	Use test set assessment to choose the most accurate and consistent ML model.	All features (including scaled continuous features)	Selection of the best model, based on F1-score, recall, and precision.
RO5	Compare confusion matrices and model False Positives (FPs) and negatives.	Diagnosis (target), All input features	Deeper insight into model reliability and real-world diagnostic utility.
RO6	To evaluate feature relevance and identify cancer prediction variables.	GeneticRisk, Smoking, CancerHistory, etc.	Ranked feature importance chart for model interpretability.
RO7	To provide an interactive Graphical User Interface (GUI) for patient data input and prediction.	User-provided inputs for all 8 features	A functional and user-friendly desktop tool for cancer risk screening.
RO8	To ensure reproducibility of results and automate performance tracking.	Full dataset + model outputs	Export of model performance metrics to Comma-Separated Values (CSV) for reproducibility and reporting.

### Contribution and novelty

**Fig. 1:**
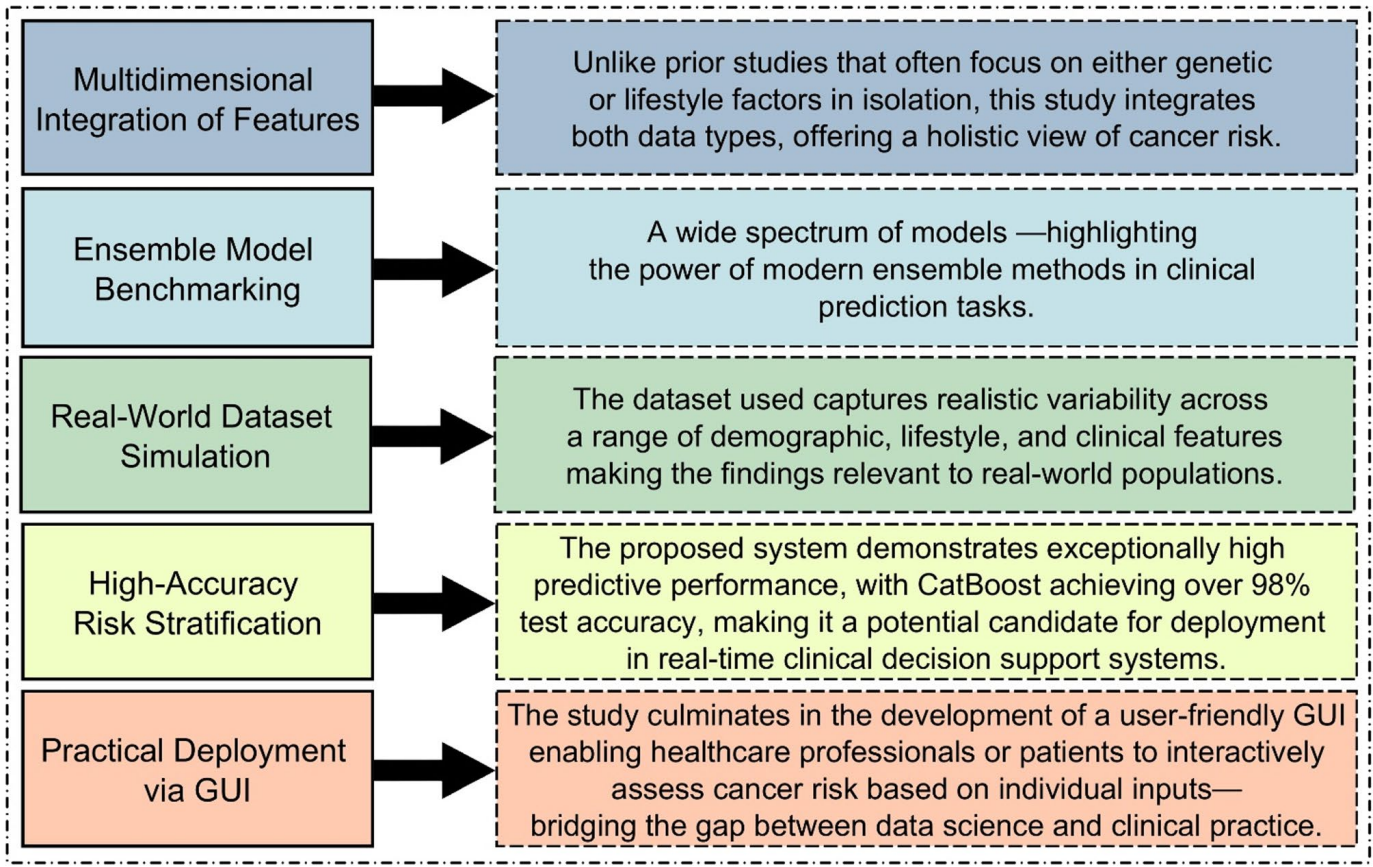
Key contributions and novel features of the proposed cancer risk prediction system.

The present study provides comprehensive ML model to predict cancer risk, incorporating both genetic susceptibility and lifestyle factors, a substantial issue that has not been adequately addressed in previous research that generally independently analyzed the associations with these features. The utilized dataset combines balanced and realistic array of patient records with different characteristics, for example age, gender, BMI, level of physical activity, smoking, alcohol consumption, the level of genetic risk and personal cancer history—providing rich basis for building more accurate risk model. By the comparative results, this research analyses one classical and ensemble ML algorithms, and demonstrates that they are capable to measure statistically and nonlinear relationships between the hardscape features. In addition to model development, the most important novelty is the implementation of a user-friendly GUI that achieves real-time prediction of cancer risk according to individual characteristics, so that both doctors and patients can be able to use it. The full workflow— from data preprocessing and model development to evaluation and GUI integration— is designed to reveal both technical depth and practical value for real-world clinical decision support, providing a scalable and flexible solution for real-world clinical decision support. Figure 1 presents the key contributions of the proposed cancer risk prediction system, highlighting its methodological and practical innovations.

Figure 1 demonstrates that the proposed system offers several significant contributions, including multidimensional feature integration, model benchmarking, real-world dataset simulation, and practical deployment through an interactive GUI—collectively augmenting its applicability to clinical cancer risk prediction.

## Methodology

### Dataset presentation

The dataset used in this study consists of 1,200 patient records were collected in an Excel file having structured labelled columns for different clinical and lifestyle data, this study used a subset of the publicly available Cancer Prediction Dataset (1,200 out of 1,500 samples), which is shared under the Attribution 4.0 International (CC BY 4.0) license^32^. Every row is an individual patient and every column relays a certain feature for risk and diagnosis of cancer. The dataset includes a total of nine features, in addition to the target variable used for prediction. A detailed description of all features and their types is provided in Table 2.

The diagnosis variable is the target of prediction in this study. It facilitates the categorization of patients according to their cancer diagnosis status. The dataset is equitably distributed among the characteristics and the target class, hence facilitating an impartial training procedure for ML models.

**Table 2:** Description of dataset features.

Feature name	Type	Description
Age	Integer	Patient’s age, ranging from 20 to 80 years
Gender	Binary	Gender of the patient (0 = Male, 1 = Female)
BMI	Continuous	Body Mass Index, ranging from 15 to 40
Smoking	Binary	Smoking status (0 = No, 1 = Yes)
GeneticRisk	Categorical	Genetic risk level (0 = Low, 1 = Medium, 2 = High)
PhysicalActivity	Continuous	Weekly hours of physical activity, ranging from 0 to 10
AlcoholIntake	Continuous	Alcohol consumption in units per week, ranging from 0 to 5
CancerHistory	Binary	Personal history of cancer (0 = No, 1 = Yes)
Diagnosis	Binary	Target variable indicating cancer diagnosis (0 = No Cancer, 1 = Cancer)

**Fig. 2:**
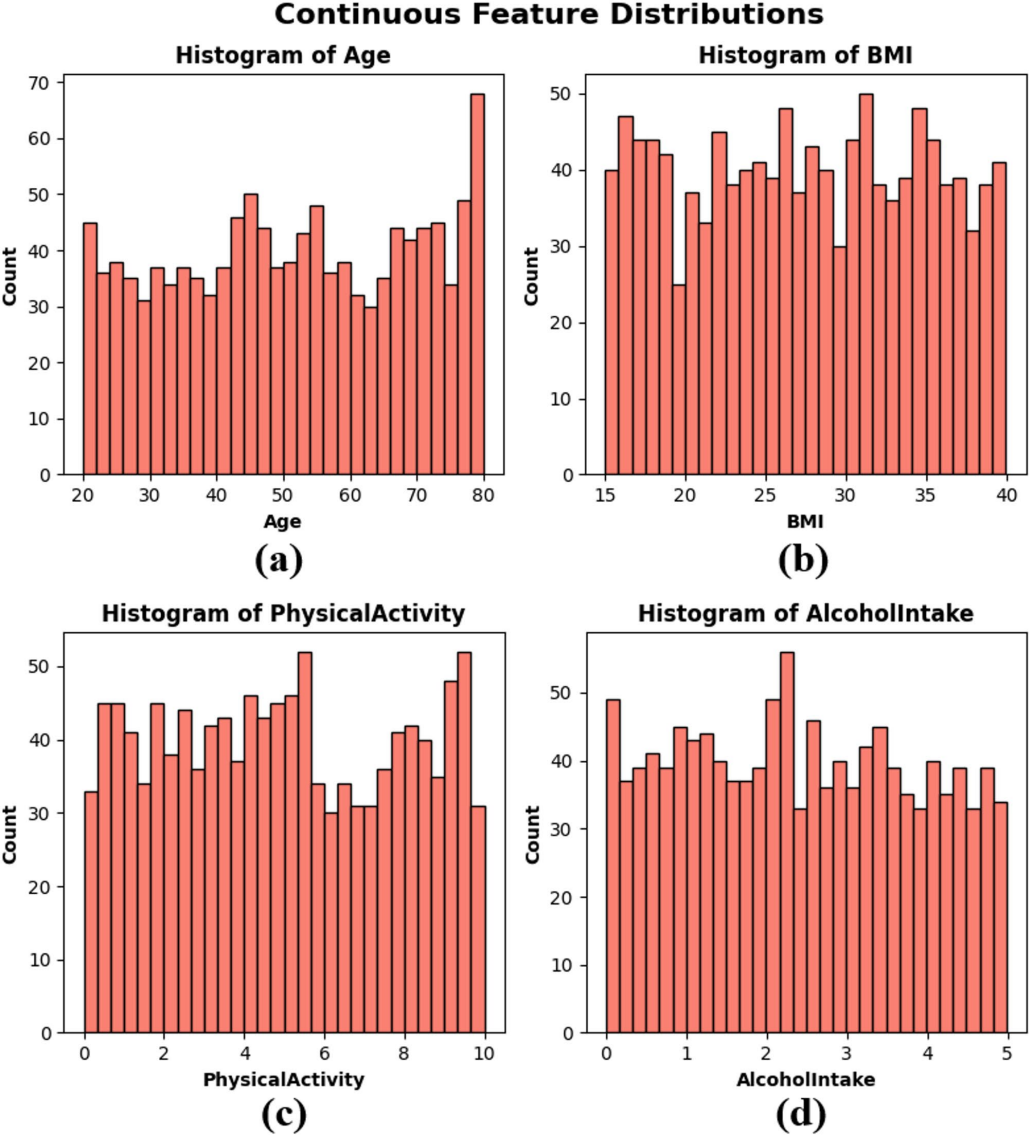
Histograms of continuous features — (**a**) Age, (**b**) BMI, (**c**) Physical activity, and (**d**) Alcohol intake.

Frequency distributions charts which are called Histograms were created of Age, BMI, Physical activity and Alcohol intake to visualize the distribution of continuous variables in the dataset. These factors represent important aspects of an individual’s health and life that could be related to cancer risk. The age distribution in subplot Fig. 2(a) is predominantly uniform across the 20 to 80 range, with a modest increase in frequency among older people. The BMI distribution in subplot Fig. 2(b) is uniformly distributed between 15 and 40, signifying a heterogeneous patient population regarding body composition. Physical activity in subplot Fig. 2(c) exhibits variability throughout the population, with significant frequencies observed across all levels from 0 to 10 h per week. The alcohol consumption depicted in subplot Fig. 2(d) has a uniform distribution ranging from 0 to 5 units, devoid of significant grouping. The histograms illustrate that the dataset exhibits authentic variability and is well-balanced, essential for constructing robust and generalizable ML models, as seen in Fig. 2.

**Fig. 3:**
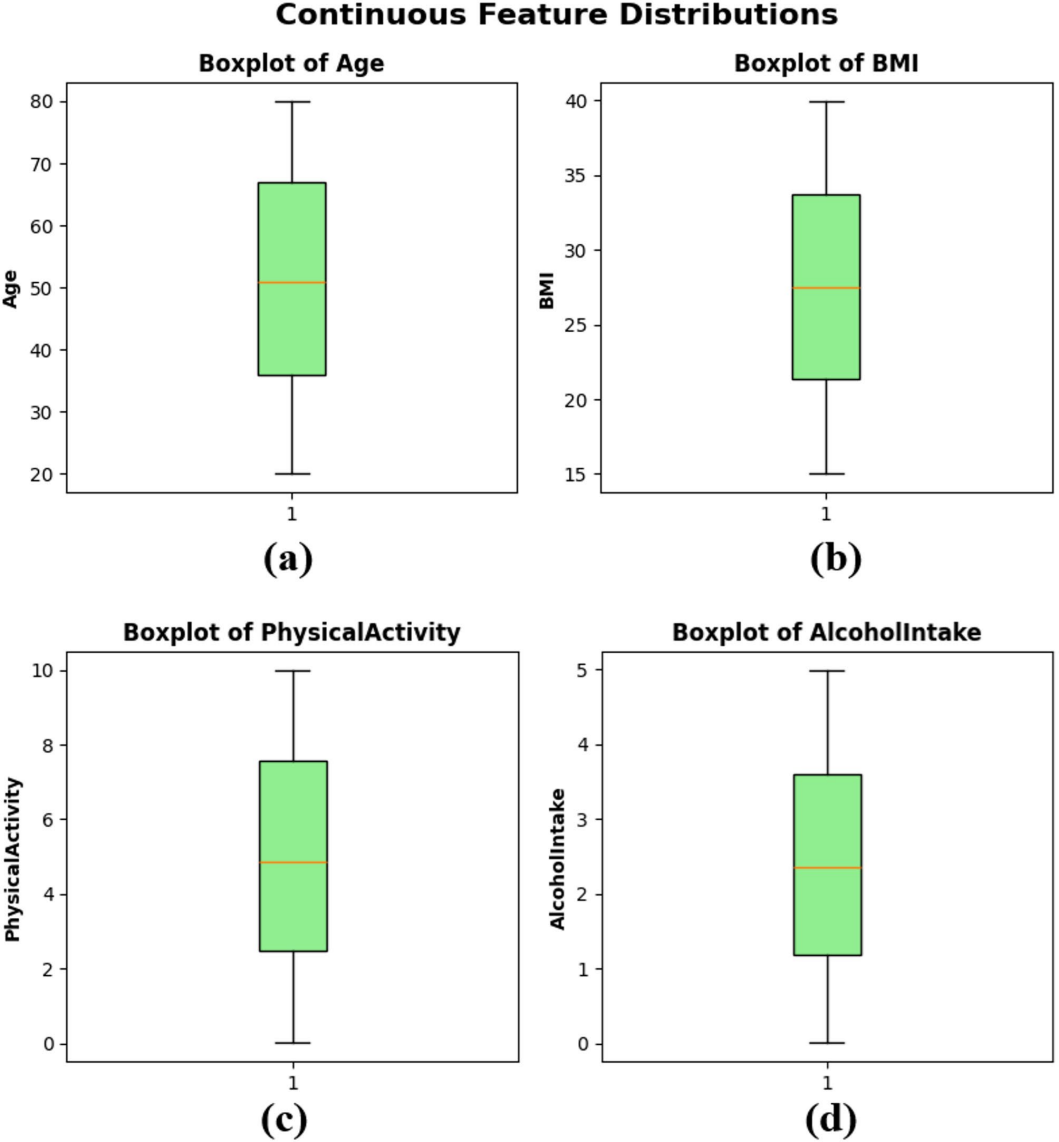
Boxplots of continuous features — (**a**) Age, (**b**) BMI, (**c**) Physical Activity, and (**d**) Alcohol Intake.

Boxplots were created to illustrate the distribution, central tendency, and possible outliers of the continuous variables in the dataset. Figure 3(a) depicts an age distribution with a median of roughly 50 years, an Interquartile Range (IQR) of about 35 to 65, and the absence of extreme outliers. The BMI feature in subplot Fig. 3(b) shows a balanced distribution, with the middle value around 27 and a narrow IQR, indicating similar body composition values among the sample. Physical activity in subplot Fig. 3(c) ranges from 0 to 10 h per week, with a median close to 5, reflecting a balanced distribution of physical activity levels among patients. Lastly, alcohol intake in subplot Fig. 3(d) also shows a wide distribution from 0 to 5 units per week, with a median slightly above 2 units. The boxplots indicate that the dataset is free of significant skewness or extreme outliers in the continuous features, supporting its suitability for training models in ML, as presented in Fig. 3.

**Fig. 4:**
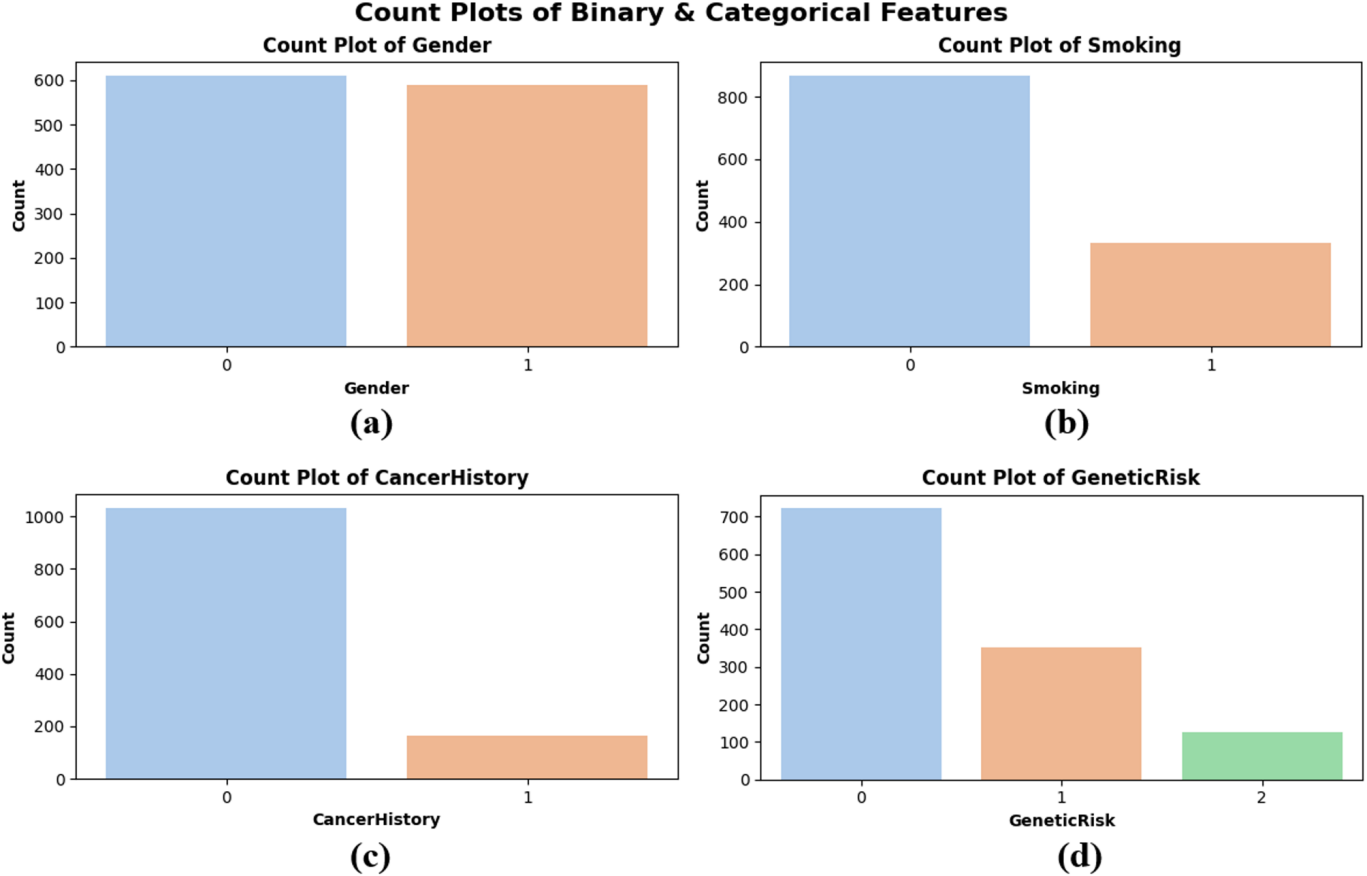
Count plots of binary and categorical features — (**a**) Gender, (**b**) Smoking, (**c**) Cancer History, and (**d**) Genetic Risk.

Count plots were created to assess the distribution of categorical and binary data, specifically for Gender, Smoking, CancerHistory, and GeneticRisk. Figure 4 illustrates that the gender distribution in subplot Fig. 4(a) is nearly balanced between males (0) and females (1), signifying equitable representation across sexes. Figure 4(b) illustrates that a greater proportion of patients are non-smokers, potentially indicating lifestyle trends among the population. In sidebar Fig. 4(c), most patients indicate no personal history of cancer, whereas a smaller nevertheless notable proportion has been previously diagnosed. The genetic risk factor in subplot Fig. 4(d) is classified into low (0), medium (1), and high (2) levels. The majority of patients are categorized as low-risk, although a smaller number are deemed high-risk. These plots illustrate the dataset’s balanced characteristics and authentic variability, confirming that the model is trained on a representative sample, as depicted in Fig. 4.

**Fig. 5:**
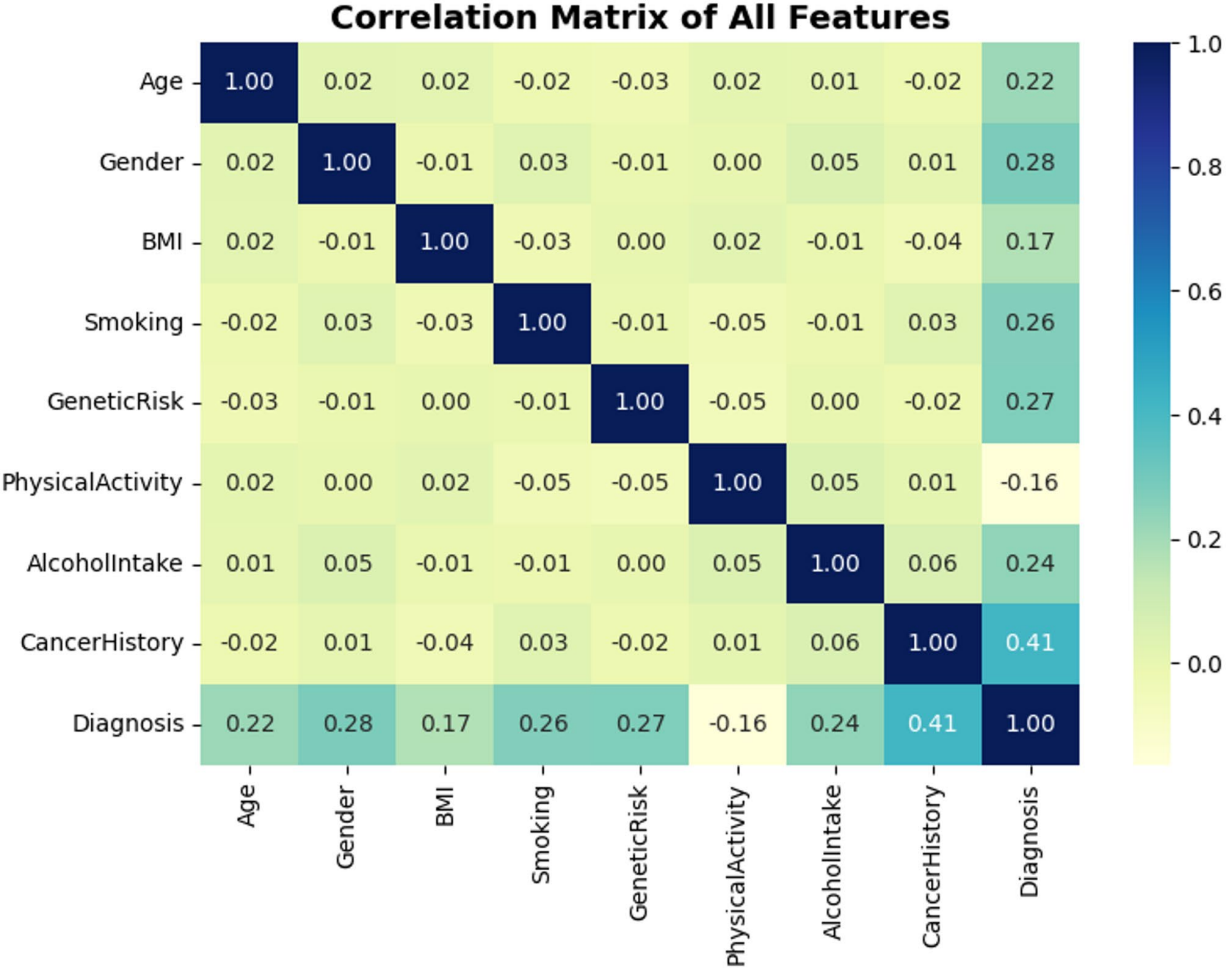
Correlation matrix of all features including diagnosis.

The matrix correlation gives statistical readings over each linear relationship between either features in the dataset and the target (Diagnosis). As can be seen from Fig. 5, CancerHistory has the highest correlation with the target and the correlation value is 0.41. This implies a mild linear relationship, or in other words, the patients who had cancer in past are more likely to have cancer in the future, as it is clinically more relevant. Other features exhibiting a high correlation with the target variable include Gender (0.28), GeneticRisk (0.27) and Smoking (0.26). While most of the feature pairs exhibit weak or negligible correlations—such as BMI and GeneticRisk or Age and PhysicalActivity—This diversity demonstrates the multi-dimensionality of the dataset, and justifies utilizing non-linear models to capture complex interactions. The above correlation observations in Fig. 5 affirm the importance of the chosen features as they are justified to have further impact in the predictive modeling.

### Workflow overview

**Fig. 6:**
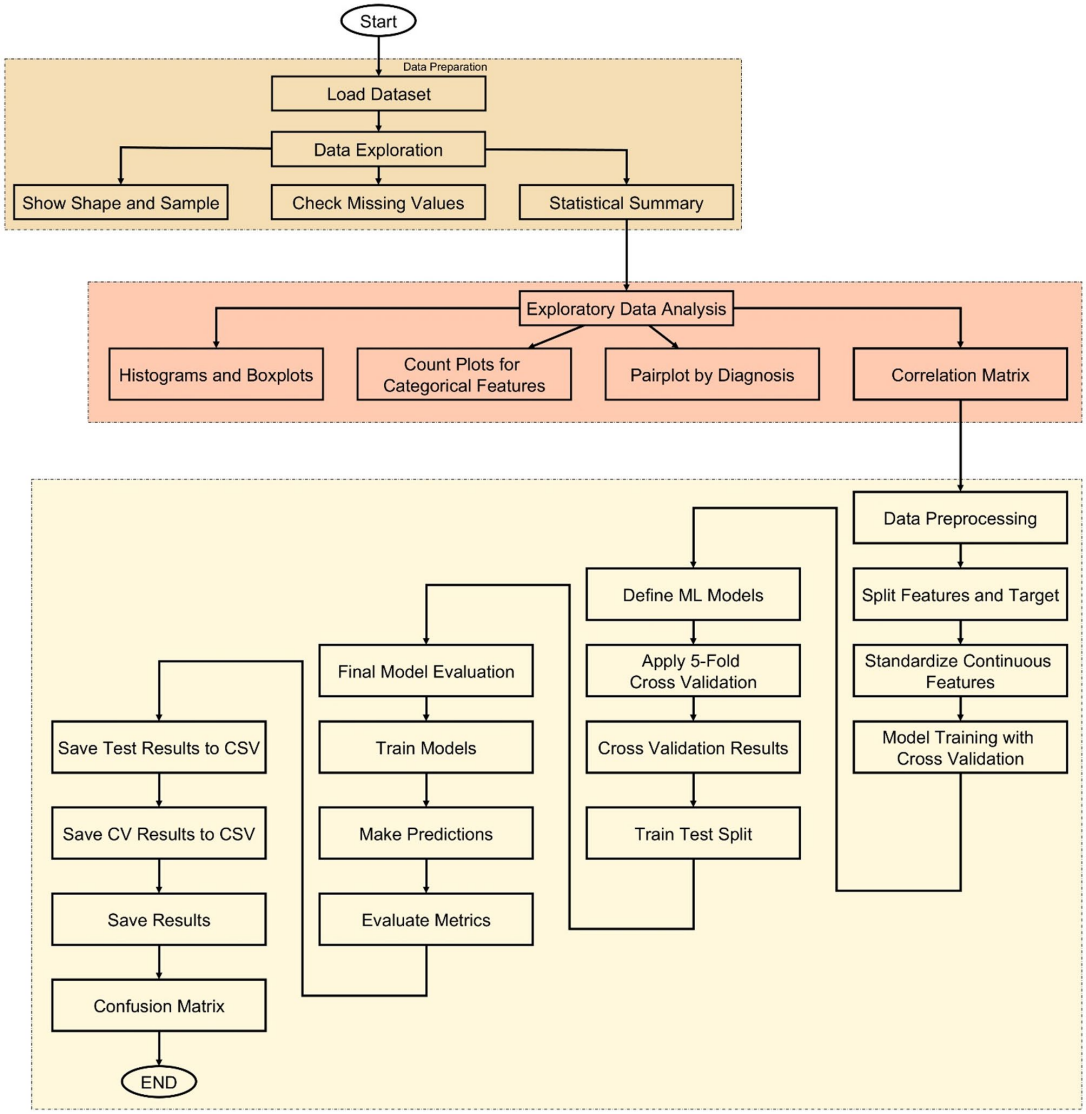
End-to-end workflow of the cancer prediction system incorporating data exploration, model training, evaluation, and GUI deployment.

The methodology of this study follows a structured and data-driven approach to develop an accurate cancer prediction system using ML, as illustrated in Fig. 6. The process begins with loading the dataset, which contains various patient features such as age, gender, BMI, smoking status, genetic risk, physical activity, alcohol intake, and personal history of cancer, along with the target variable, diagnosis. After loading, the dataset undergoes thorough exploration to understand its structure and integrity. Basic information such as shape, sample rows, and missing values is examined, followed by statistical summaries that provide insight into the distribution of each feature. To deepen this understanding, EDA is conducted through visualizations including histograms, boxplots, and count plots, helping to identify patterns and anomalies. A correlation matrix is also generated to detect relationships among numeric variables.

Following exploration, data preprocessing is performed where the dataset is split into input features and the target label. Continuous features are standardized using StandardScaler to ensure uniform scaling across models. The core of the methodology lies in training multiple ML models—such as Logistic Regression (LR), Decision Tree (DT), RF, Gradient Boosting (GB), Support Vector Machines (SVMs), k-Nearest Neighbors (k-NN), CatBoost, eXtreme Gradient Boosting (XGBoost), and Light Gradient Boosting Machine (LightGBM)—using 5-fold stratified cross-validation. This ensures balanced and robust evaluation by assessing each model’s accuracy across different data splits. The model with the highest average cross-validation accuracy is selected as the best-performing model.

To further validate model performance, a train-test split is applied and all models are evaluated on unseen test data. Key metrics such as accuracy, precision, recall, and F1-score are calculated for each model, and confusion matrices are plotted to visualize their classification performance. All evaluation results are saved as CSV files to support reproducibility and reporting. Finally, a GUI is developed using Tkinter, allowing users to enter patient information and receive instant predictions from the trained model. This end-to-end workflow ensures that the system is not only accurate and reliable but also accessible and user-friendly for practical use.

### Evaluation metrics

In order to verify the efficacy of the developed ML models, some popular classification metrics were adopted. These measures help in having a full overview of the efficiency of each model in separating patients into those with and without the disease, a very important aspect in all the medical- related prediction tasks.

One of the indicators is in particular the accuracy which is defined as percentage of correctly classification predictions (both negative and positive) over all the predictions performed. This provides a general sense of model correctness, as presented in Eq. (1). However, accuracy alone may not be sufficient in medical datasets, particularly when the cost of FNs or FPs is high.

To gain deeper insight, precision and recall were also evaluated. Precision measures the proportion of True Positive (TP) cases among all positive predictions, which is crucial to minimize false alarms and avoid subjecting healthy individuals to unnecessary concern or follow-up procedures, as shown in Eq. (2). On the other hand, recall—also known as sensitivity—focuses on the ability of the model to correctly identify actual cancer cases, ensuring that as few positive cases as possible go undetected. This is calculated as described in Eq. (3). As there is usually a trade-off between precision and recall, this study used the F1-score to have a single performance score that balances both. This takes the harmonic mean of precision and recall, and is useful when there is a slight class imbalance or when FPs and FNs are both concerning. Mathematically, the F1-score is defined in Eq. (4).

Alongside these scalar metrics, a confusion matrix was created for each model to illustrate the quantities of TPs, True Negatives (TNs), FPs, and FNs. This facilitated a clear comprehension of the model’s performance across various prediction types.

Each model was initially assessed utilizing a 5-fold stratified cross-validation approach, which maintained the class distribution within each fold. This yielded a strong assessment of the model’s generalization ability. Subsequently, the models were assessed on a distinct 20% hold-out test set, and identical metrics were calculated to gauge their real-world predictive efficacy.

1 $$\:Accuracy=\frac{TP+TN}{TP+TN+FP+FN}\:$$

2 $$\:Precision=\frac{TP}{TP+FP}\:$$

3 $$\:Recall\:\left(Sensitivity\right)=\frac{TP}{TP+FN}\:$$

4 $$\:F1-Score=\frac{Precision\:\times\:\:Recall}{Precision\:+\:Recall}\:$$

## Results

Cross-validation and independent test set evaluation were performed to assess the discriminatory performance of several ML models to predict risk of cancer given lifestyle and genetic data. The results also show the differences of accuracy, precision, recall and F1-score of the models when comparing each other.

As shown in Table 3, CatBoost outperformed with the highest mean cross-validation accuracy (0.9850) while XGBoost and GB performed closely behind, with 0.9742 and 0.9733, respectively. In addition, both of these models showed impressive stability, with small standard deviations in cross-validation accuracy, meaning that they perform consistently in most of the folds. In contrast, the mean accuracies for k-NN and LR were lower at 0.8292 and 0.8742 (mean accuracy values for k-NN and LR with higher variation) with these models struggling to generalize well to this particular area of medical classification even more so due the limited complexity of these models.

**Table 3:** Cross-Validation performance of machine learning models.

Machine learning model	Mean cross-validation accuracy	Standard deviation of cross-validation accuracy
k-NN	0.8292	0.0137
LR	0.8742	0.0165
SVM	0.9017	0.0162
DT Classifier	0.9133	0.0113
RF Classifier	0.9500	0.0139
LightGBM	0.9675	0.0143
GB Classifier	0.9733	0.0057
XGBoost	0.9742	0.0055
**CatBoost**	**0.9850**	**0.0068**

Subsequent analysis of the test dataset validated the enhanced efficacy of ensemble-based models. Table 4 demonstrates that CatBoost attained the best test accuracy of 0.9875, alongside flawless precision (1.0000) and a notable F1-score of 0.9820. XGBoost, LightGBM, and GB demonstrated exceptional performance, with a test accuracy of 0.9750 and F1-score (0.9643). Conversely, conventional models such as LR and k-NN exhibited inferior F1-scores of 0.8046 and 0.8188, respectively, highlighting the superiority of modern GB approaches in this classification problem.

These findings highlight the power of ensemble learning approaches, particularly boosting-based algorithms, in modelling the complex, non-linear relationships often present in health-related datasets to improve the robustness of cancer risk prediction systems.

**Table 4:** Performance metrics of machine learning models for cancer risk prediction based on lifestyle and genetic data.

Model	Test accuracy	Test precision	Test recall	Test F1-score
LR	0.8583	0.7865	0.8235	0.8046
k-NN	0.8875	0.9531	0.7176	0.8188
SVM	0.9250	0.8941	0.8941	0.8941
DT	0.9333	0.9059	0.9059	0.9059
RF	0.9667	0.9753	0.9294	0.9518
GB	0.9750	0.9759	0.9529	0.9643
XGBoost	0.9750	0.9647	0.9647	0.9647
LightGBM	0.9750	0.9759	0.9529	0.9643
** CatBoost**	**0.9875**	**1.0000**	**0.9647**	**0.9820**

**Fig. 7:**
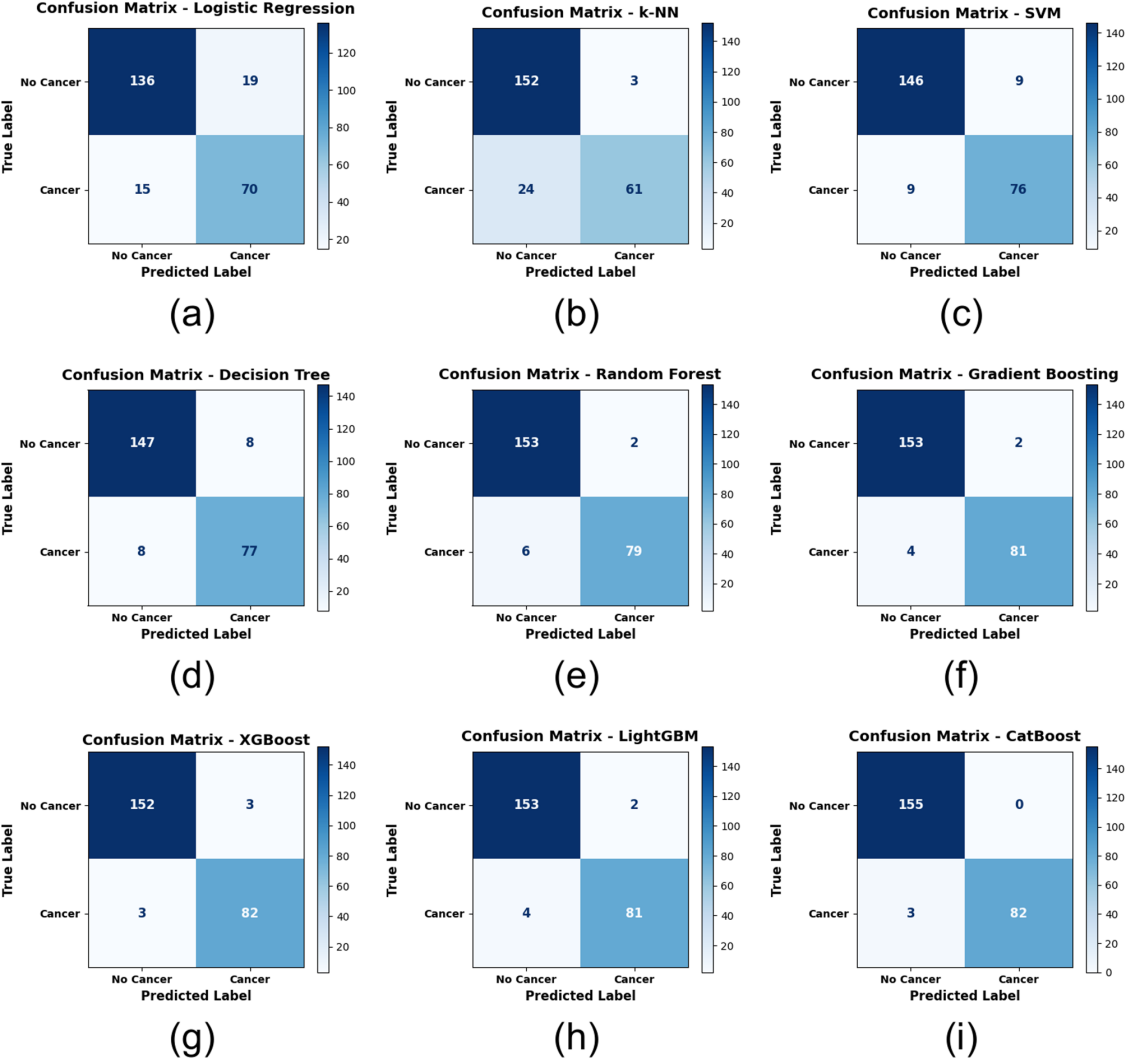
Confusion Matrices of Machine Learning Models — (**a**) Logistic Regression, (**b**) k-Nearest Neighbors, (**c**) Support Vector Machine, (**d**) Decision Tree, (**e**) Random Forest, (**f**) Gradient Boosting, (**g**) XGBoost, (**h**) LightGBM, (**i**) CatBoost.

To better understand the classification results for cancer and non-cancer case scenarios, the performance of individual ML models was investigated with respect to the confusion matrices. The confusion matrices scatter plots shown in Fig. 7 present the distribution of the four components of classification across all models True Positive (TP), TN, FPs and FN. In contrast, more straightforward models such as LR and k-NN, illustrated in Fig. 7(a) and Fig. 7(b) respectively, exhibited significantly higher misclassification rates, particularly in terms of FNs, which represent a critical error in medical diagnostics. Figure 7(a) illustrates that LR erroneously classified 15 cancer cases as non-cancer, whereas Fig. 7(b) reveals that k-NN misclassified 24 cancer cases.

Conversely, ensemble-based models exhibited markedly enhanced categorization performance. The DT depicted in Fig. 7(d) and the RF illustrated in Fig. 7(e) exhibited reduced misclassifications, recording only 8 and 6 FNs, respectively. GB, XGBoost, and LightGBM, illustrated in Fig. 7(f), Fig. 7(g), and Fig. 7(h), each misclassified fewer than five instances of cancer. Among all models, CatBoost, illustrated in Fig. 7(i), attained the best classification accuracy, recording zero FPs and merely three FNs—underscoring its potential reliability in critical medical screening contexts.

Figure 7 illustrates the enhanced efficacy of boosting-based ensemble models in accurately classifying both positive and negative cancer cases, hence reducing diagnostic errors that may result in significant clinical repercussions.

## Deployment and future work

### Model deployment as API or clinical tool

The concept of the proposed system is designed to maintain the deployment in practical scenarios either on desktop or web to help deploy this real world applicable cancer risk prediction system. Though the current model is only exposed to a Tkinter-based GUI for local predictions, the framework is entirely malleable for introducing as a RESTful Application Programming Interface (API), following recent conventions in Flask or FastAPI. Although this flexibility enables easy integration with Hospital Information Systems (HIS), Electronic Medical Records (EMRs) and mobile health applications. To use the model, clinicians would remotely input de-identified patient data using secure HyperText Transfer Protocol (HTTP) requests and receive real-time cancer risk assessments.

Deployment in a clinical setting would include several key elements. There are a couple of advantages to this approach — first, the entire ML pipeline from data preprocessing to feature scaling to prediction logic can be defined in a single Docker container, isolated from the host environment enabling reproducible performance across hosts. The hosted model, either in a secure cloud capability like Amazon Web Services (AWS) or Azure, or in local healthcare infrastructure, can be made accessible securely and at scale via API endpoints. Data privacy and access control are important, so HyperText Transfer Protocol Secure (HTTPS) encryption and authentication, using JSON Web Tokens (JWT), can be applied to protect the API, through Health Insurance Portability and Accountability Act (HIPAA) or General Data Protection Regulation (GDPR).

A simple web interface using frontend technologies like React or some interactive framework like Streamlit can be built to make it easier for the healthcare professionals to use. The proposed system could feature a dashboard for clinicians to enter patient data and model predictions, and interpretability tools such as SHapley Additive exPlanations (SHAP) values for how important each feature was to the particular prediction. Overall, this deployment strategy provides for the accessibility, reliability, and scalability of the model extending its uptake to actual, real-time, clinical workflows, and reaching beyond the research setting.

### Model monitoring and retraining

Once a ML model has been deployed with clinical applications, the long-term maintenance of that model is essential, since model degradation can happen as time goes by and the underlying data (data drift), patient composition (population drift) or medical practice changes. Deployment entails continuous monitoring and periodic retraining of the cancer prediction model to maintain accuracy and ensure safety.

Prediction through the API can be logged with clinician feedback and ideally confirmation of true diagnoses to monitor performance. This provides an actual audit trail that facilitates looking back to the performance. researchers can use some tools, like Alibi Detect or Evidently AI that have already pre-built tests for distributional changes in the input/features you are providing or the prediction outcome, or they can also use their own statistical tests (like calculating shifts in mean and variance). The model should be re-evaluated at regular intervals (e.g. quarterly) using the latest available patient data, to ensure that its performance measures such as accuracy, recall and F1-score are still within clinically viable limits.

If decline or drift of performance occurs, retraining incrementally using a mixture of older and newly labelled data can be performed. Data Version Control (DVC) can be used to keep track of data version, and corresponding pipelines can be automated via continuous integration and delivery Continuous Integration/Continuous Deployment (CI/CD) tools, such as GitHub Actions or Jenkins to ease this process. In order to provide all the quality of transparency and compliance with the regulatory guidelines, a model registry containing detailed metadata: version of the training data, performance measurement, and approval should be kept.

These monitoring and retraining strategies ensure that the system is not just technically sound and clinically reliable, but also adheres to best practices in Machine Learning Operations (MLOps). It guarantees the functionality and safety of the cancer prediction model when the model moves through research prototype to a reliable tool in day-to-day medical applications.

## Conclusion

Through the analysis of both genetic predispositions and lifestyle-related factors, this study demonstrates the potential of ML in predicting cancer risk. Nine supervised learning algorithms were trained and assessed using test set performance measures and cross-validation using a dataset of 1,200 patient records. Of these ensemble methods, CatBoost, XGBoost, and GB showed higher performance than the others in accuracy, recall, precision, and F1-score. CatBoost is the best performing model, achieving test accuracy and F1-score of 98.75% and 0.9820 respectively. This study confirms that integrating genetic predisposition indicators with modifiable lifestyle factors—such as smoking, BMI, and physical activity—significantly enhances cancer prediction accuracy compared to using genetic risk alone. Analysis of feature importance indicated that cancer history, genetic predisposition, and smoking were the most significant predictors. These discoveries correspond with established clinical knowledge and underscore the significance of data-driven methodologies in medical risk evaluation. In conclusion, this study illustrates that ML models, particularly ensembles based on boosting are suitable for scalable and patient-friendly cancer risk prediction. Larger and more heterogeneous datasets may be helpful in future research as well as adding further clinical characteristics to make the prediction more accurate and to ensure generalizability of the model.

## Supplementary Information

Below is the link to the electronic supplementary material.

Supplementary Material 1

## Data Availability

All data generated or analysed during this study are included in this published paper. The custom code is provided in the supplementary materials. Additional data supporting the findings of this study are available from the corresponding author upon reasonable request.

## Abbreviations

AI: Artificial Intelligence
API: Application Programming Interface
AWS: Amazon Web Services
BMI: Body Mass Index
CatBoost: Categorical Boosting
CI/CD: Continuous Integration / Continuous Deployment
CSV: Comma-Separated Values
DT: Decision Tree
DVC: Data Version Control
EDA: Exploratory Data Analysis
EMRs: Electronic Medical Records
FN: False Negative
FP: False Positive
GB: Gradient Boosting
GDPR: General Data Protection Regulation
GUI: Graphical User Interface
HIPAA: Health Insurance Portability and Accountability Act
HIS: Hospital Information Systems
HTTP: HyperText Transfer Protocol
HTTPS: HyperText Transfer Protocol Secure
IQR: Interquartile Range
JWT: JSON Web Tokens
k-NN: k-Nearest Neighbors
LightGBM: Light Gradient Boosting Machine
LR: Logistic Regression
ML: Machine Learning
MLOps: Machine Learning Operations
RF: Random Forest
SHAP: SHapley Additive exPlanations
SVM: Support Vector Machine
TN: True Negative
TP: True Positive
XGBoost: eXtreme Gradient Boosting
